# Attempts to preserve and visualize protein corona on the surface of biological nanoparticles in blood serum using photomodification

**DOI:** 10.3762/bjnano.15.130

**Published:** 2024-12-30

**Authors:** Julia E Poletaeva, Anastasiya V Tupitsyna, Alina E Grigor’eva, Ilya S Dovydenko, Elena I Ryabchikova

**Affiliations:** 1 Institute of Chemical Biology and Fundamental Medicine, Siberian Branch of Russian Academy of Science, Lavrent’ev av., 8, Novosibirsk, 630090, Russian Federation

**Keywords:** chylomicrons, extracellular vesicles, lipoproteins, photomodification, protein corona

## Abstract

A protein corona is present on any nanoparticle (NP) entering biological fluids; however, the existence of a natural protein corona on natural NPs has not been experimentally confirmed. We used our previously developed photomodification method to fix the natural corona on “biological nanoparticles” (bio-NPs) in fetal bovine serum and newborn bovine serum; native sera served as a control. To isolate photomodified bio-NPs, we used ultracentrifugation (UC), sucrose gradient (12%, 30%, and 50%), and sucrose cushion (30%) methods. Isolated bio-NPs were visualized using transmission electron microscopy. The results showed that a protein corona is present on extracellular vesicles and lipoproteins isolated by UC. The isolation of bio-NPs through sucrose gradient or cushion did not preserve the protein corona. At the same time, we observed signs of a negative effect of the sucrose gradient on bio-NPs of intact and photomodified serum; when isolating these particles on a sucrose cushion, no negative effects were observed. We believe that the data we present will be useful to researchers using sucrose solutions to isolate bio-NPs and working on the properties of the protein corona. In this work, we have obtained direct images of a “natural” protein corona on natural bio-NPs of blood serum for the first time

## Introduction

The existence of a protein corona on all nanoparticles (NPs) entering biological fluids, confirmed by hundreds of publications, has become a paradigm of modern nanoscience. The extent of research on the properties of the protein corona is growing, and new data are appearing regarding the influence of the corona and its changes on various processes in the body, including pathological ones. The efforts of many researchers are aimed at finding approaches and methods for “managing” the composition of the protein corona, which, in particular, can provide the possibility of targeted delivery of drugs [1–3].

The protein corona is formed by two layers on any NP, called the hard and soft coronas. The former is tightly linked the surface of the NPs and is stable when isolating the NPs, which allows for determining its protein composition [4–7]. In contrast, the components of the soft corona are weakly bonded to the underlying hard one and are easily separated by the slightest force. The vacated space is quickly occupied by neighboring components of the medium, as a result of which the composition of the soft corona is constantly changing, even when a NP suspension is stirred [8–9].

As data accumulate on the role of the protein corona in the implementation of nanomedicine, researchers are increasingly interested in studying the protein corona on extracellular vesicles (EVs), mainly exosomes, which play an important role in the transmission of molecular signals in the body. The influence of the protein corona on EVs on their interaction with body cells, including cells of the immune system, and the role of the corona in the pathogenesis of chronic diseases are being studied [5,10–13]. A number of works are devoted to the study of protein corona formation on EVs previously isolated from biological fluids [14–15].

The existence of a “natural” protein corona on EVs in the body is assumed, but no studies have been published directly showing the presence of a “natural” protein corona on EVs. The presence or absence of a “natural” corona on EVs is certainly of interest to researchers. In connection with the topic of the “natural” protein corona, it is appropriate to note that, in addition to EVs, other “natural” NPs are present in the blood, namely, lipoproteins (LPs), which are not vesicles. The content of LPs in blood is incomparably higher than that of EVs [5,16]. Previously, we detected LPs using transmission electron microscopy (TEM) in various biological fluids (tears, urine, ascitic fluid, and nutrient media collected after culturing various cell lines) [17–21]. We consider the term “biological nanoparticles” (bio-NPs) proposed by Jens B. Simonsen and Rasmus Münter [22] to be adequate in defining the totality of all types of EVs and LPs present in the blood, and we will use this term in the present work.

We used fetal bovine serum (FBS) and newborn bovine serum (NBS) to study the “natural” protein corona on bio-NPs. We suppose that a protein corona naturally exists on the surface of bio-NPs and may be lost during the isolation process. To prevent the corona loss, we fixed it on the surface of the bio-NPs by the photomodification method. We developed this method recently for fixing a full protein corona on model NPs with lipid envelope. One of the proofs of protein corona formation on the particle surface was its visualization using TEM [23]. This study motivated us to try to fix and visualize the protein corona on “natural” serum bio-NPs, which also have a lipid surface.

Fixation of the full protein corona currently is considered as a reliable method to analyze the protein composition of the corona. For the first time, we applied photomodification to obtain NPs bearing a full protein corona on the lipid surface, the proteins of which could be identified by mass analysis [23]. Hossein Mohammad-Beigi and colleagues published another approach in 2020 [7]. They used methods of click chemistry to fix proteins on the surface of silica and polystyrene NPs and thereby obtained the full corona. In principle, the effect of click chemistry and photomodification is the same. Both fix the full corona; however, the methods differ in details. When click chemistry is performed, soft corona proteins bind only to pre-modified hard corona proteins, whereas photomodification additionally captures “free” soft corona proteins that are not bound to the hard corona. In this way, photomodification provides a more complete representation of soft corona proteins, which are then identified by mass analysis methods.

**Figure 1:**
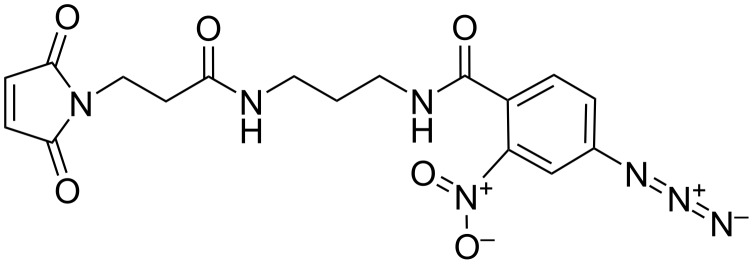
Chemical formula of the photoactivatable cross-linker 4-azido-*N*-[3-[3-(2,5-dioxopyrrol-1-yl)propanoylamino]propyl]-2-nitrobenzamide (PACL).

The main component in the photomodification process is a photomodifier, the choice of which was described in detail and justified earlier. The best option was the photoactivatable cross-linker 4-azido-*N*-[3-[3-(2,5-dioxopyrrol-1-yl)propanoylamino]propyl]-2-nitrobenzamide (PACL), which was synthesized in the Laboratory of Organic Synthesis (ICBFM SB RAS, Novosibirsk, Russia) [23]. PACL (Figure 1) is a molecule carrying two orthogonal reactive groups, namely, (i) a maleimide residue that modifies serum proteins at their thiol groups and (ii) an nitroaryl azide group to form covalent cross-links between proteins and nanoparticles under the influence of UV radiation. When incubated in the dark, PACL binds to the thiol groups of serum proteins (i.e., it modifies them). The reaction mixture is then irradiated with UV, under the action of which the nitroaryl azide group covalently fixes the modified proteins on the surface of the NPs. This mechanism has been demonstrated for model NPs [23], and we believe that it will also operate in the serum protein–bio-NP system.

The photomodification method made it possible to obtain a protein corona on the surface of model NPs incubated with FBS, fix it, and visualize it. Particular attention was paid to the study of the ability of blood serum proteins to bind to each other under the influence of PACL and subsequent UV irradiation. However, if was found that serum proteins bind only to the surface of model NPs and do not cross-link with each other. In other words, in FBS incubated with PACL and irradiated with UV, the formation of serum protein aggregates was not detected, PACL specifically interacted with the NP surface. It is interesting that we observed the attachment of LPs to the surface of the protein corona on model NPs [23].

**Figure 2:**
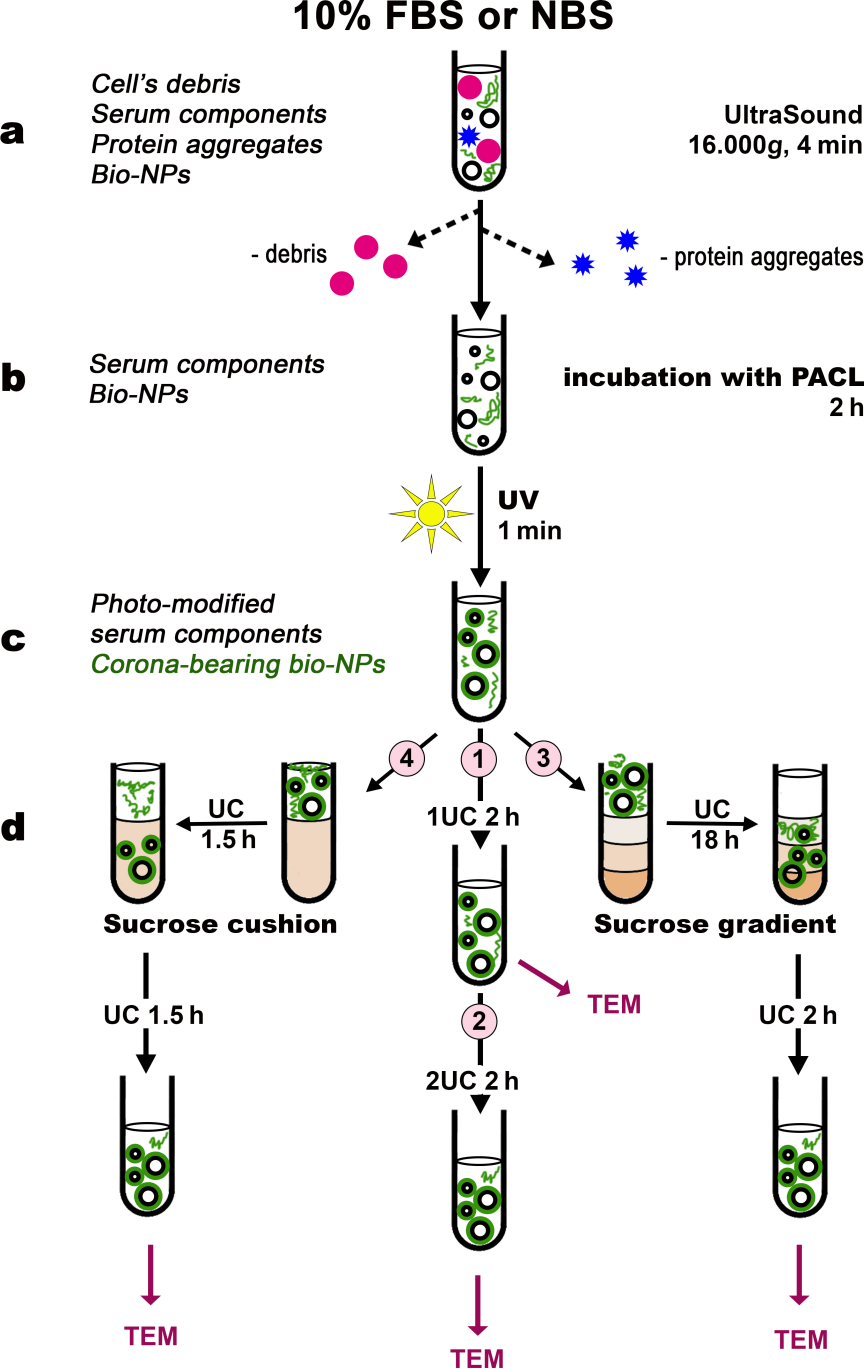
Experimental scheme for isolating bio-NPs from sera. (a) To remove protein aggregates and cellular debris, serum samples were sonicated (4 min) and centrifuged at 16,000*g* (4 min, 25 °C). (b) The supernatant was collected and incubated with the photomodifier PACL. (c) UV irradiation (310 nm, 8.2 mW/cm^2^, 1 min). Unmodified serum samples served as a corona-free control. (d) Bio-NPs were isolated using the following methods: (d1) ultracentrifugation (UC) (2 h, 100,000*g*); (d2) double UC (2 h, 100,000*g*); (d3) UC in a sucrose density gradient of 12%, 30%, and 50% (18 h, 100,000*g*) followed by UC to remove sucrose (2 h, 100,000*g*); and (d4) UC on a 30% sucrose cushion (1.5 h, 100,000*g*) followed by UC to remove sucrose (1.5 h, 100,000*g*). The obtained corona-bearing bio-NPs and control samples were visualized in TEM.

We supposed that the incubation of sera with PACL followed by UV irradiation (photomodification) could result in the fixation of serum proteins originally bound to the bio-NPs’ surface, that is, the fixation of the “natural” protein corona. We did not isolate bio-NPs beforehand, instead we worked with solutions of native sera. Figure 2 shows the workflow of our experiments for obtaining bio-NPs from FBS and NBS.

## Results and Discussion

### Isolation of bio-NPs by ultracentrifugation

**Figure 3:**
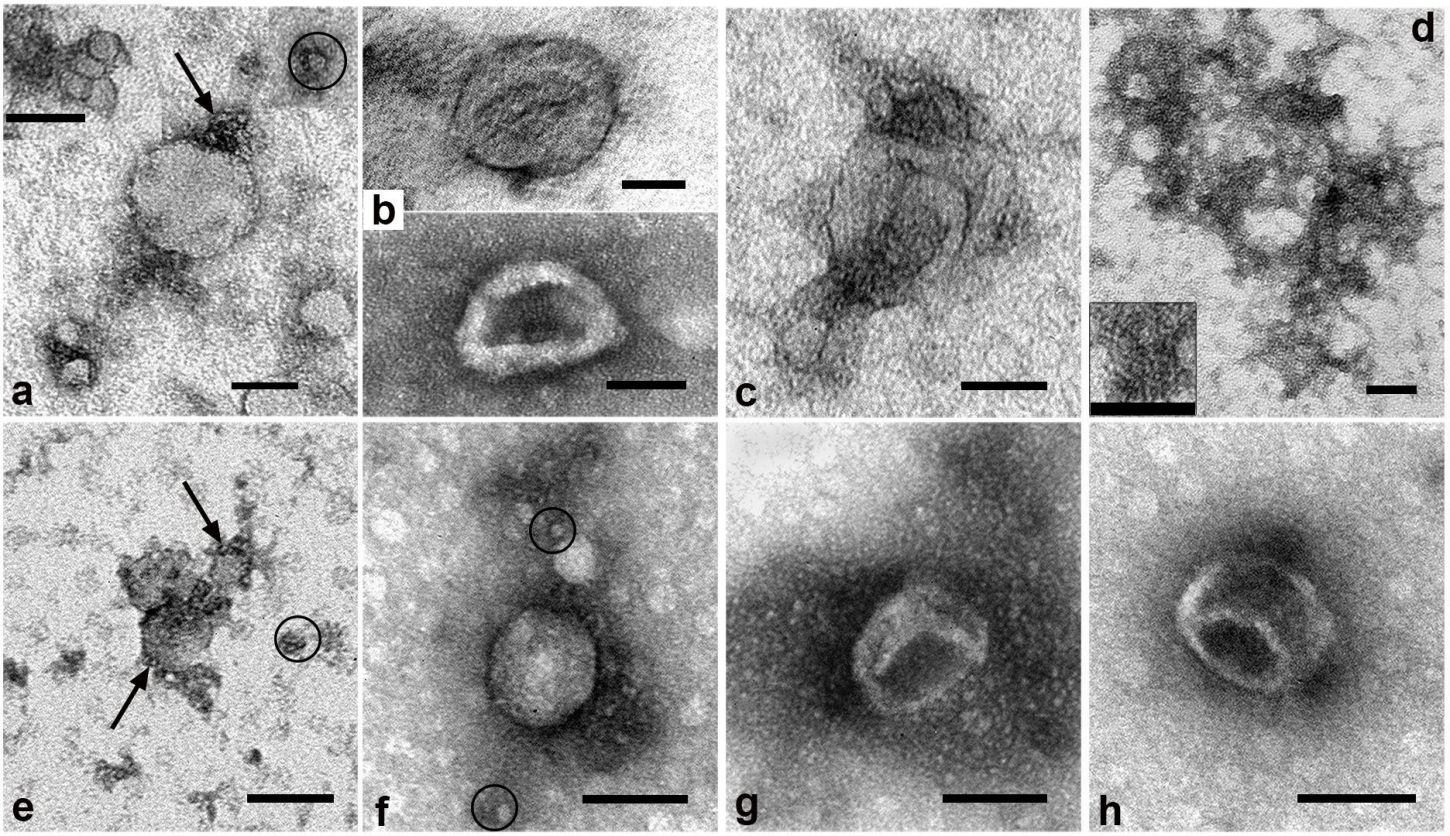
Representative images of bio-NPs isolated from 10% FBS by single (top row) or double (bottom row) UC. (a,e,f) LPs; circles show high-density LPs (≤10 nm). (a,e) Minute spherical particles are shown with arrows. (b,c,g,h) EVs. (d) Structureless serum components. TEM, negative staining with 0.5% uranyl acetate. The length of the scale bars corresponds to 50 nm.

To obtain samples of intact bio-NPs, we used single or double ultracentrifugation (UC) of 10% FBS, and the resulting samples were negatively stained and examined in TEM. The largest share of bio-NPs observed in these samples were spherical LPs of low electron density, having a homogeneous structure and different sizes (Figure 3a,e,f). According to [24], we identified LPs with a diameter of 10 nm and less as high-density LPs (Figure 3a,e,f), particles of 20–30 nm as low-density LPs (Figure 3a,e,f), and particles of 40–80 nm as very low-density LPs (Figure 3a). Clusters of tiny spherical particles were often observed on the surface of many LPs (Figure 3a,e).

The content of EVs in samples isolated from FBS by UC was incomparably lower than the content of LPs. The majority of EVs were 60–120 nm in size, resembled collapsed balls (Figure 3b,g,h), and were morphologically consistent with exosomes. Large vesicles (120–180 nm) were rare and usually deformed (Figure 3c). The surface of the EVs and LPs often carried structureless serum components.

Accumulations of structureless and grainy serum components of different electron density, sizes and shapes, and without clear boundaries, which gave the grids a contaminated appearance, were observed in the samples (Figure 3d). Some FBS components were visualized as white points (about 1 nm), often arranged in short chains that formed branches on the grid (Figure 3d, insert). The presence of contaminants on the grid and on the surface of bio-NPs made it difficult to visualize the latter after single UC. In the samples obtained by double UC, bio-NPs were scattered on the grids mainly individually and were the same types of bio-NPs as after single UC (Figure 3e–h). Double UC did not result in a significant decrease of sample contamination. But it did result in a noticeable decrease in the number of bio-NPs, the surface of which, however, became a little cleaner. Loss of target components after double and triple UC of blood serum or plasma has been noted previously [24–25].

Bio-NP samples of 10% NBS were prepared similarly to those from FBS by single and double UC and were examined in TEM. Both kinds of samples contained identical types of bio-NPs, with a prevalence of LPs. Structureless NBS components contaminated the surface of grids and bio-NPs, and the degree of contamination was visually higher than in the case of FBS. We repeated the isolation of bio-NPs by UC from FBS and NBS eight times and found that neither single nor double UC provided bio-NPs with a clean surface; the particles were always associated with a greater or lesser amount of contaminants. Thus, single and double UC for 2 h at 100,000*g* did not remove all serum components bound to the surface of bio-NPs.

**Figure 4:**
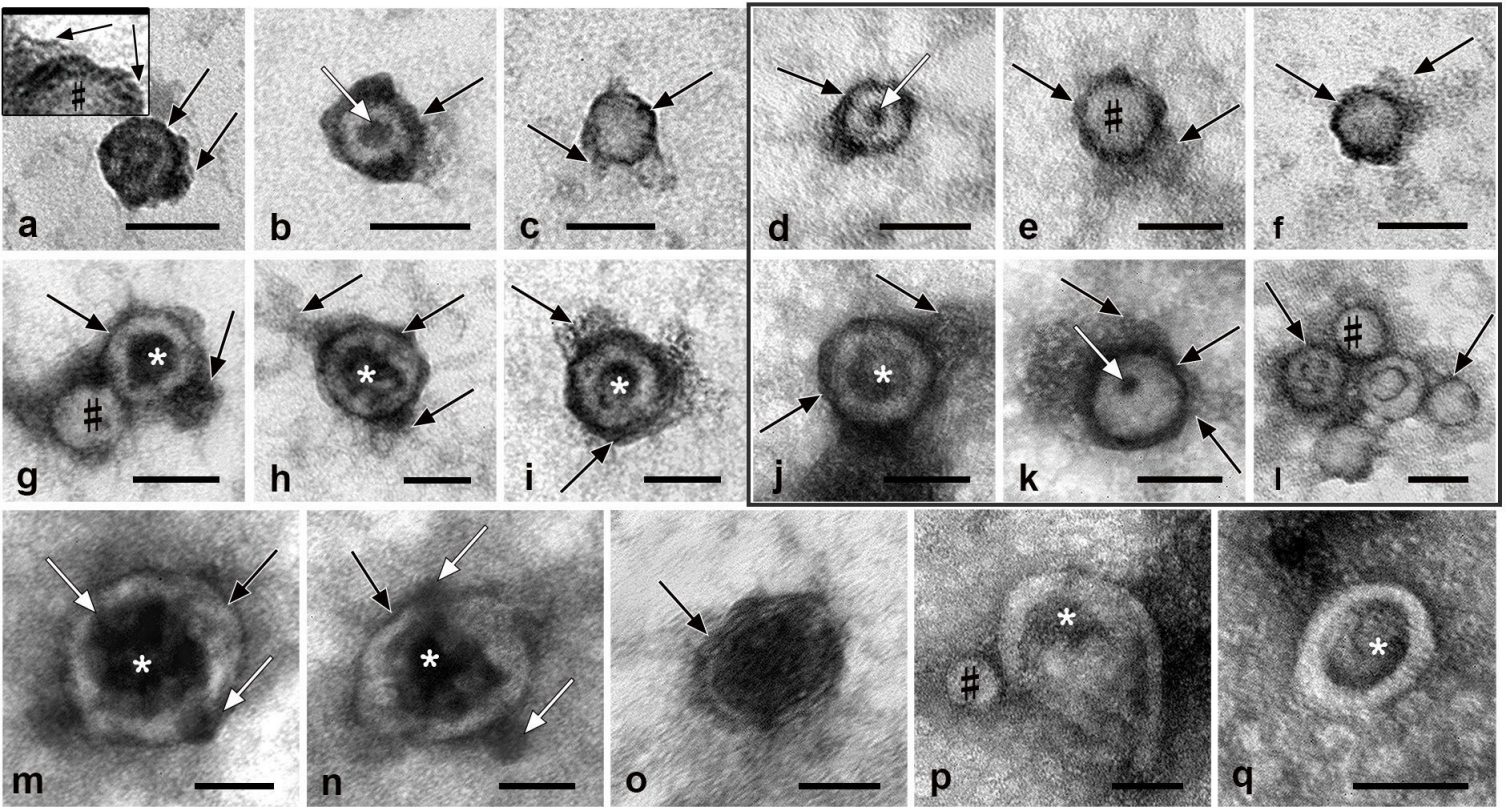
Representative images of bio-NPs isolated by UC from photomodified 10% FBS and NBS (shown in the frame) samples. The upper row shows 30–40 nm bio-NPs, and the middle row shows 50–70 nm bio-NPs. (m–o) EVs 60–120 nm in size. (p, q) EVs and LP devoid of an electron-dense layer. The protein corona on the surface of bio-NPs is shown by the black arrows; an enlarged fragment of corona is shown on the insert in (a); white arrows show globules; (*) indicates deepenings in the bio-NPs; (#) shows examples of a “body” of bio-NPs. TEM, negative staining with 0.5% uranyl acetate. The length of the scale bars corresponds to 50 nm.

The next stage of our work was to study the effect of sera photomodification on the ultrastructure of bio-NPs isolated from FBS and NBS by single or double UC. Significant changes in the ultrastructure of bio-NPs comparing with native sera were detected (Figure 3 and Figure 4).

Incubation of FBS and NBS with PACL and subsequent UV irradiation resulted in the appearance of an electron-dense material adsorbed directly onto the bio-NPs’ surface (Figure 4a–o). The material appeared homogeneous, and its high electron density made it difficult to study its fine structure. The material continued, without a visible boundary, into an appendage with polymorphic structure. The appendage showed rounded protrusions, tails, and clouds formed by structureless material of variable electron density without clear boundaries. Sometimes, fine-grained components of serum with medium electron density were recognized in the appendage. Thus, photomodification of FBS and NBS resulted in the appearance of an additional envelope surrounding the bio-NPs, in which an electron-dense material adjacent to the surface of the particle is clearly seen, which continues into the appendage. The thickness of this envelope significantly exceeded the thickness of the bio-NP membranes, and the shape varied greatly. In the control samples of FBS and NBS, isolated from native sera in the same way, signs of this additional envelope were absent (Figure 3). This gives us the basis to identify the envelope on bio-NPs as a protein corona fixed by the photomodification.

The observed high polymorphism of the protein corona is obviously related to the known dependence of the protein corona composition on the surface composition of bio-NPs, which are quite diverse in blood serum [1–2,26]. The plane of sorption of bio-NPs on the grid may make some contribution to their appearance. We observed a large number of different morphological types of bio-NPs bearing a protein corona, which could be of irregular shape, fragmentary, and of different thickness. On the surface of some bio-NPs, homogeneous globules (10–15 nm) were found (Figure 4b,d,k,m,n), the electron density of which was higher than that of LPs (Figure 4p). Several neighboring particles could have a joint corona, sometimes enclosing LPs and EVs (Figure 4g,l).

The bio-NPs in the photomodified samples had a body of average electron density, the substance of which looked slightly non-homogenous. The LPs resembled tennis balls (Figure 4a–f), while the EVs, which in their native state had liquid contents, were deformed and resembled collapsed balls with a deepening. The deepening was often filled with an electron-dense substance; in this case, the EV had the appearance of a thick ring on the grids (Figure 4g–j,m–o). The analysis of the structure and identification of bio-NPs in samples obtained from photomodified sera were complicated by the changes caused by this procedure. Globules (10–15 nm) were adsorbed in the center of many LPs, giving them an appearance that is not typical for particles of this type (Figure 4b,d,k). The presence of larger, often electron-dense, structures in the center of the LPs suggested the presence of a deepening (Figure 4g–j), which is typical for EVs. However, these bio-NPs did not resemble compressed balls as the EVs, and we identified them as LPs changed in the process of photomodification.

Interestingly, bio-NPs without a protein corona (Figure 4p,q) accounted for at least half of all particles in the photomodified samples. Apparently, a number of bio-NPs have surface properties that prevent PACL from binding to them. Given the huge variety of bio-NPs with different surface structures, this fact is not surprising, but it deserves attention. In further development of the photomodification method, it will undoubtedly be useful to identify the mechanisms of this phenomenon.

**Figure 5:**
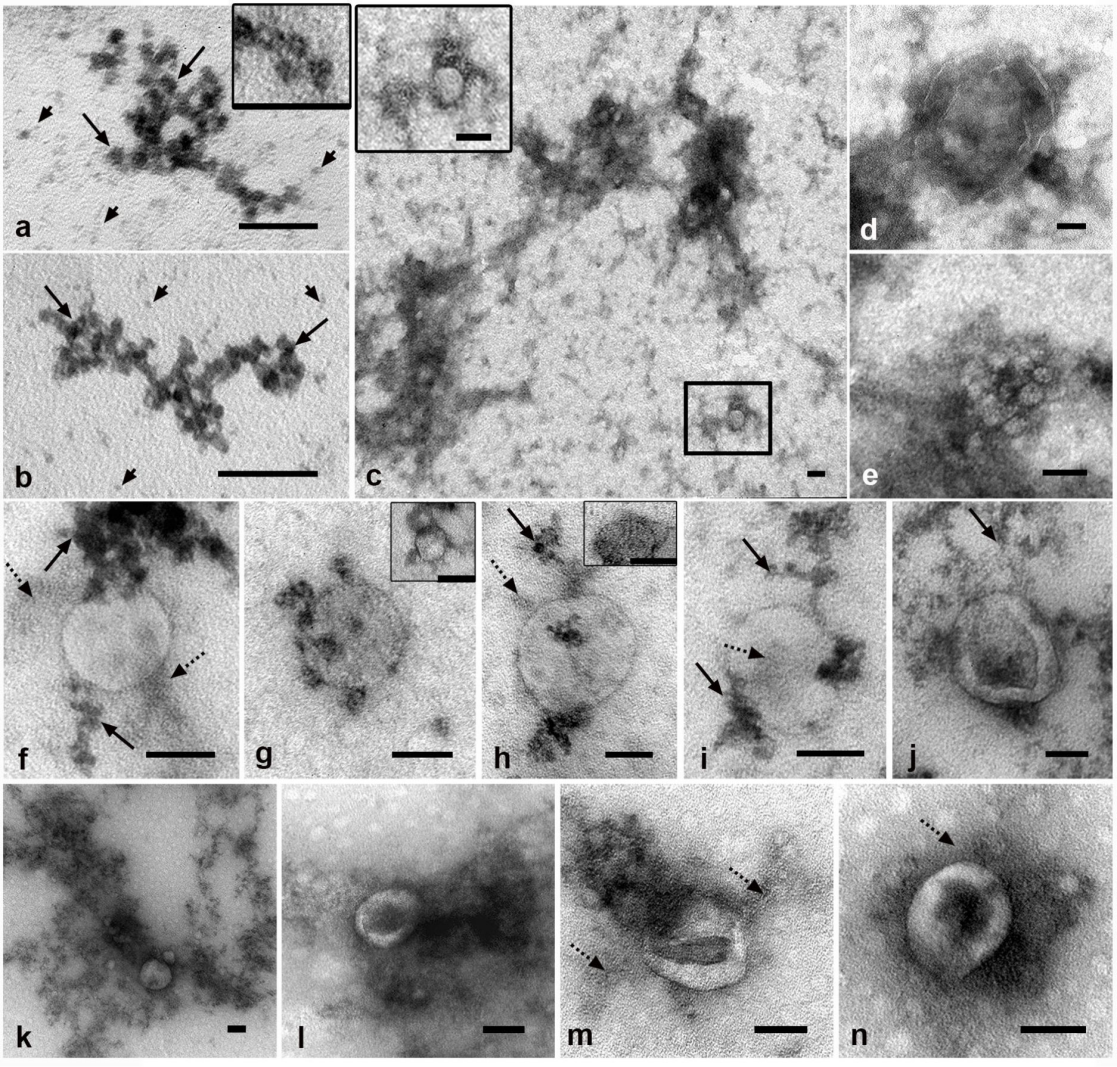
(a, b) Representative images of individual sucrose particles, clusters, and shapes in the 30% sucrose fraction after UC. The short arrows show sucrose particles, and the long arrows show clusters. (c–n) Representative images of structures in samples obtained by UC of FBS or NBS in a sucrose gradient. (c–e) Deposits of structureless serum material and bio-NPs in the 12% sucrose fraction; an enlarged LP (frame) is shown in the insert. (f–j) Bio-NPs decorated with sucrose clusters (arrows) and fine-grained material (dashed arrows) in the 30% sucrose fraction. LPs of 40–60 nm are presented in the inserts. Note the minute particles associated with the surface of the LPs in (g). (k–n) Large deposits of sucrose clusters masking bio-NPs in the 50% sucrose fraction. The dashed arrows show fine-grained material. TEM, negative staining with 0.5% uranyl acetate. The length of the scale bars corresponds to 50 nm.

It was impossible to assess the proportion of EVs or different LPs carrying a protein corona in samples obtained by UC because of the presence of serum contaminants that can mask bio-NPs. Isolation of bio-NPs from sera by UC did not result in samples fully suitable for TEM studies, and we decided to apply sucrose gradient isolation to improve the sample quality. We first studied the ultrastructure of sucrose in 12%, 30% and 50% solutions treated similarly to bio-NPs to distinguish sucrose particles from serum components. This experiment made it possible to verify that pure sucrose solutions of different concentrations that underwent UC contained particles visible in TEM (Figure 5a,b). Images of these particles were obtained for their identification in samples containing bio-NPs. Single sucrose particles were small (1–2 nm), rounded in shape, and of medium electron density; most of them were arranged in spherical clusters (5–10 nm) with electron-transparent or electron-dense centers. In turn, the clusters were connected into chains, forming various shapes (Figure 5a,b). The structure of sucrose particles and clusters were the same in the solutions of different concentrations.

We then examined the distribution of bio-NPs isolated from native 10% FBS or NBS in the sucrose gradient. Concerning the 12% sucrose fraction, the grids were heavily contaminated with loose accumulations of structureless serum components, sucrose clusters were rare. The content of bio-NPs was low, and they were immersed in the serum components (Figure 5c–e). Thus, samples isolated in the 12% sucrose fraction were not suitable for TEM studies.

The 30% sucrose fraction contained the largest number of bio-NPs and was virtually free of serum contaminants. Sucrose clusters associated with the surface of bio-NPs were frequently observed (Figure 5f–j). A fine-grained material of medium electron density was detected on some bio-NPs isolated from NBS (Figure 5f,h,i). The samples provided a clean background in the grids, which allowed us to analyze the structure of EVs and LPs, as well as to differentiate sucrose clusters and serum components. Thus, the bio-NP samples in the 30% sucrose fraction were of a quality suitable for TEM analysis and were used as a reference sample in the study of bio-NP samples isolated from photomodified sera.

In the 50% sucrose fraction, the content of bio-NPs was significantly lower than in the 30% fraction, and the bio-NPs were immersed in accumulations of sucrose clusters. Large vesicles (120–150 nm) were found in these samples in addition to other bio-NPs. The surface of the bio-NPs was often associated with fine-grained material (Figure 5k–n). The samples of the 50% sucrose fraction were virtually free of serum components; however, they were not suitable for TEM because of the small content of bio-NPs and the abundance of sucrose clusters.

The obtained results showed that purification using a sucrose gradient allows for obtaining bio-NP samples (in the 30% fraction) of sufficiently high concentration and low degree of contamination. The quality of these samples was much better than that of samples obtained by UC. It should be noted that sucrose particles were firmly bound to the surface of the bio-NPs and remained there even after additional UC for 2 h.

Passing through the gradient and staying in 30% sucrose solution noticeably changed the ultrastructure of the bio-NPs, which looked different from those after UC alone, that is, the vesicle membranes were thinner, and the surface and contour of the LPs were somewhat less definite. These changes were not measurable, but they were visually noticeable (compare Figures 3–5), especially when directly viewing the samples in TEM. The bio-NPs exhibited less features, and many of the smallest dots that form the three-dimensional image had disappeared. We were unable to find published data on the effect of sucrose on the ultrastructure of bio-NPs (as well as artificial lipid NPs), and there is no information on the crystallization of sucrose during the UC process. We can suppose that long-term (18 h) exposure to 30% sucrose solutions under UC resulted in the separation of some components from the surface of bio-NPs, probably due to the mechanical action of sucrose particles and clusters on the surface of bio-NPs. It is also possible to suppose chemical interactions of sucrose molecules with surface molecules of bio-NPs. However, we do not have factual data for such speculations, and additional research is needed here. Sucrose gradient is widely used for bio-NP isolation, the details of the method vary considerably [27–30], and 30% solution is often used. However, no one has studied the effect of this method of isolation on the ultrastructure of bio-NPs. We also did not set such a goal, but observing the effect of sucrose, we decided to draw the attention of researchers to this fact. Indeed, such action of sucrose can distort the results of bio-NP studies, especially regarding surface structures.

**Figure 6:**
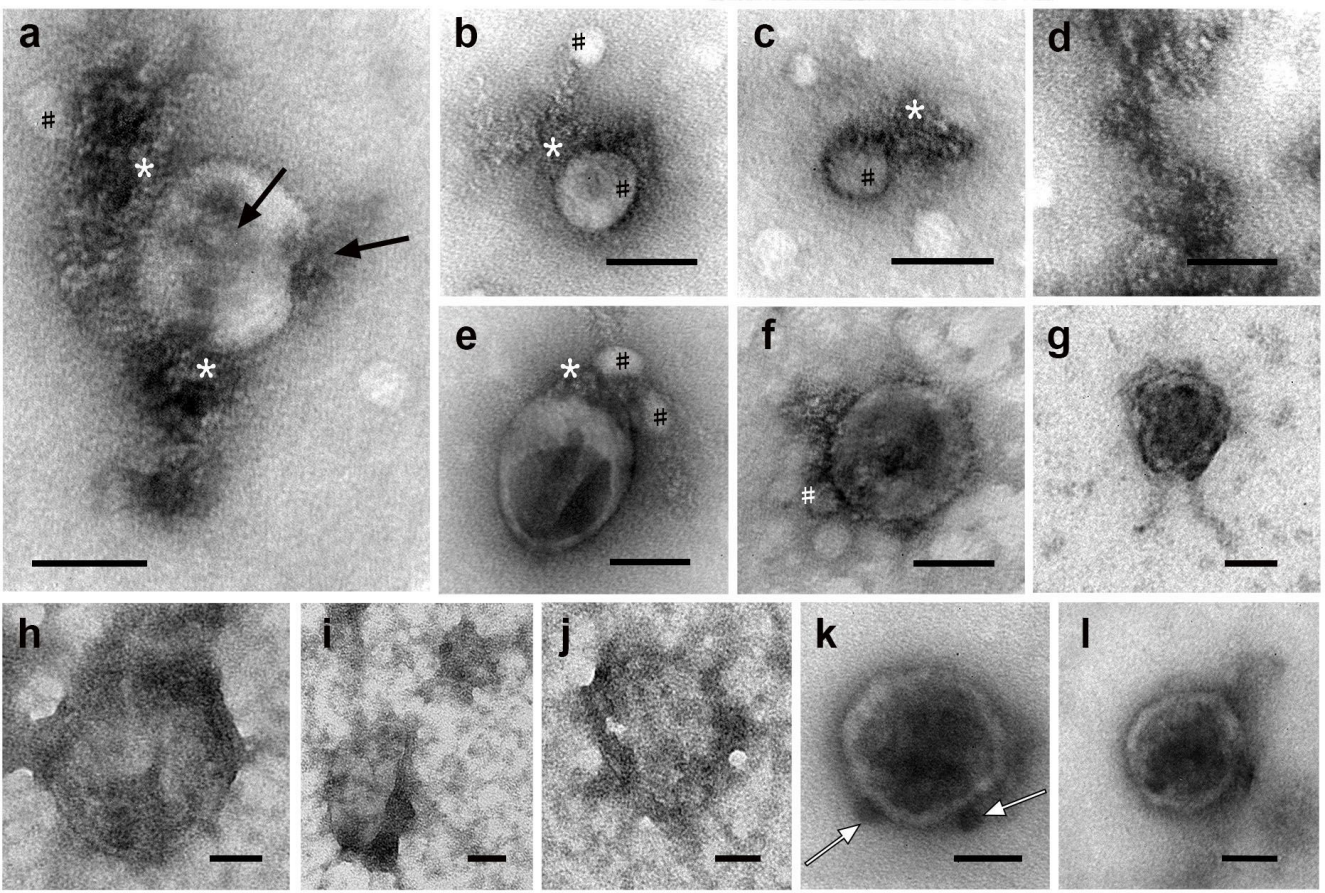
Representative images of bio-NPs isolated on sucrose gradient (30 % fraction) after photomodification of FBS (a–g) and NBS (h–l). (a–c) LPs. (d) Free accumulation of electron-transparent particles. (e, f) EVs; (*) accumulations of electron-transparent particles; (#) LPs; arrows show dust-like substance. (g) Chylomicron particle. (h–j) heavily polluted bio-NPs and (k, l) chylomicrons in NBS. White arrows show globules on chylomicron surface. TEM, negative staining with 0.5% uranyl acetate. The length of the scale bars corresponds to 50 nm.

The next step of our study was to examine the ultrastructure of bio-NPs isolated from photomodified sera using a sucrose gradient. Primary screening in TEM showed that the 30% sucrose fraction contained the highest number of bio-NPs, as did the samples obtained from native sera. Samples isolated from FBS showed a clear background in TEM, and the structureless components of serum were rare and small. We discovered a picture that was fundamentally different from the samples isolated from FBS by UC alone. The protein corona on the surface of bio-NPs, which was clearly visible on bio-NPs isolated by UC (Figure 4), was absent on the surface of bio-NPs from the 30% sucrose fraction (Figure 6). In place of a corona, loose accumulations of small (2–3 nm) polymorphic electron-transparent particles on the surface of both EVs and LPs were observed (Figure 6a–c,e,f). These particles accumulated in the form of a tail (Figure 6a–c) or a cap (Figure 6e,f), with most of the surface remaining clear. The same kind of particles not associated with bio-NPs were also present in the samples (Figure 6d). Such particles were not detected in any bio-NP sample isolated from FBS by UC alone, and neither in the control samples prepared on a sucrose gradient. A dust-like substance of medium electron density, which formed accumulations of different sizes and shapes (Figure 6a) was associated with the surface of bio-NPs isolated after photomodification. We observed the association of EVs with LPs (Figure 6e,f) in samples of bio-NPs isolated on a sucrose gradient. An unexpected finding in the FBS-derived samples were rare chylomicrons that appeared deformed (Figure 6g). Fine changes in ultrastructure noted in control samples isolated on a sucrose gradient (see above) were also detected after photomodification. For example, the loosening of a LP surface is clearly seen in Figure 6a,c,f.

We were unpleasantly surprised by the quality of the samples isolated on a sucrose gradient from photomodified NBS. Having discovered unacceptable contamination of the grids (Figure 6h–j), we repeated the isolation four times, but did not obtain samples of the required quality. The observed contamination originated from NBS; sucrose clusters were not detected. However, despite the heavy pollution, the content of bio-NPs was comparable to that of the FBS samples. The particles were scattered individually, and in some the membrane could be seen (Figure 6h–j). In these same polluted samples, chylomicrons were observed (Figure 6k,l), the content of which was higher than in the samples obtained from FBS. Chylomicrons were located in small areas of the grid that were free of contamination. They had a low-electron-density envelope, to which globules (Figure 6k) and accumulations of a dust-like substance (Figure 6l) were associated. Chylomicrons were observed after all four repetitions of the isolation of bio-NPs on a sucrose gradient from NBS, and the phenomenon of their separation from the surrounding contaminations is unclear. The chylomicrons were identified based on published data regarding their size and morphology [31–34]. The experiments on isolating bio-NPs from photomodified FBS and NBS did not achieve the goal to isolate protein corona-bearing bio-NPs. However, we revealed a few interesting facts regarding the interaction of bio-NPs with sucrose, as well as the emergence of chylomicrons with the ability to repel serum components.

**Figure 7:**
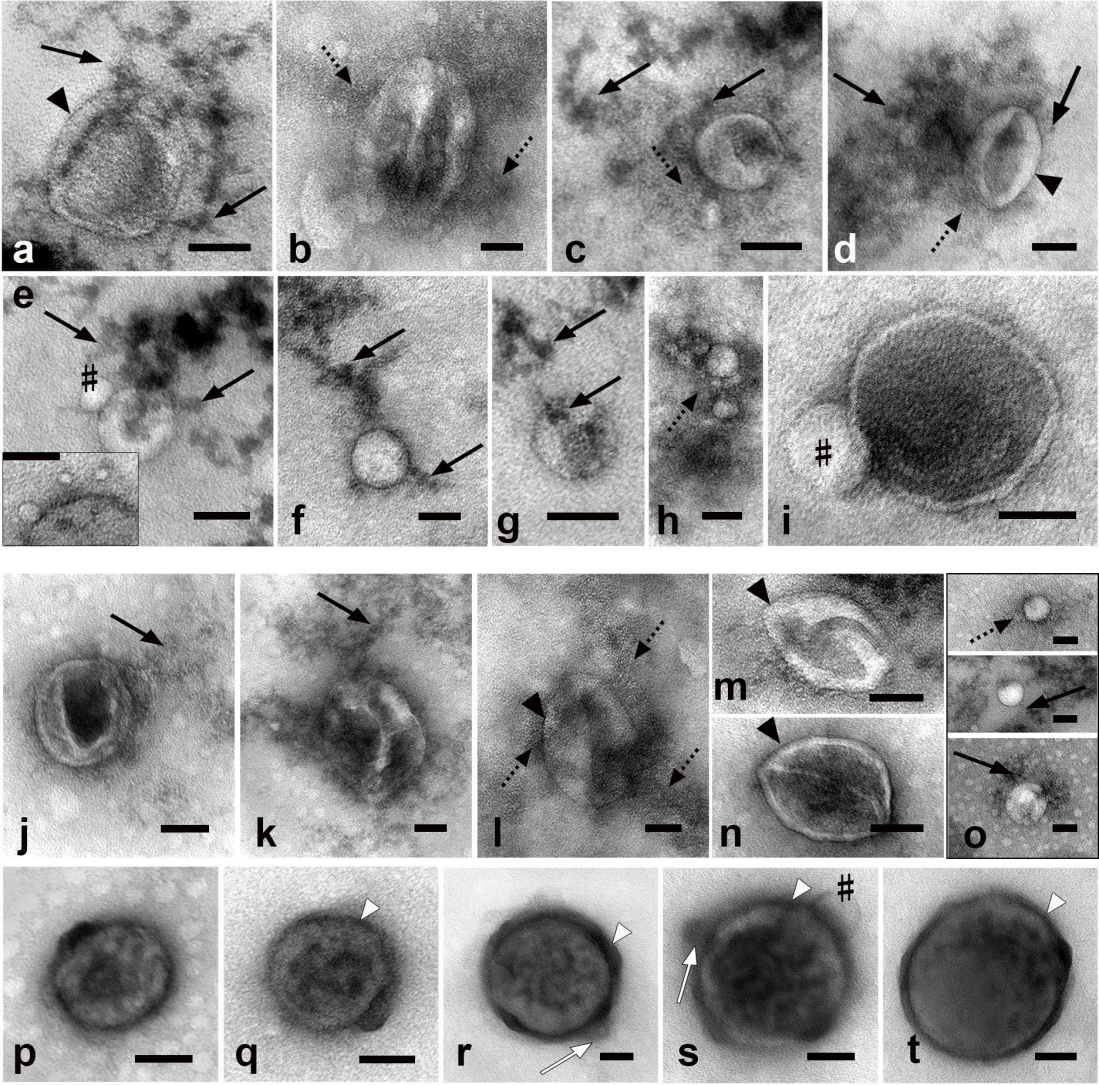
Representative images of bio-NPs isolated from native FBS and NBS (two upper rows) and after sera photomodification (two bottom rows) using purification on a 30% sucrose cushion. (a–d, j–n) EVs. (e–h, o) LPs, the insert in (e) shows LPs of 10 nm. (i, p–t) Chylomicrons. LPs associated with bio-NPs are designated with (#). The arrows show sucrose clusters; the dotted arrows show the dust-like substance; the arrowheads show the membrane envelope of EVs; the white arrowheads show the protein corona (electron-dense layer) on the surface of chylomicrons. TEM, negative staining with 0.5% uranyl acetate. The length of the scale bars corresponds to 50 nm.

The gradient purification conditions we used were quite harsh (18 h of UC in 30% sucrose), and we set up another experiment, namely, the purification of intact and photomodified FBS and NBS samples on a sucrose cushion (30% sucrose, 90 min UC). The use of the sucrose cushion purification [35] made it possible to obtain samples with a high content of bio-NPs of all types, including chylomicrons, from intact FBS and NBS (Figure 7a–i). The EVs, LPs, and chylomicrons had a typical appearance, and their surface (except for the chylomicrons) was polluted with sucrose clusters (Figure 7a,c–g); some bio-NPs were associated with a dust-like substance (Figure 7b–d).

Isolation of bio-NPs on the sucrose cushion allowed us to obtain samples with a relatively high content of bio-NPs, devoid of serum contaminants. Numerous sucrose clusters were in contact with the bio-NPs. There were no changes in the ultrastructure of bio-NPs as those noted in the samples isolated on sucrose gradient. This observation demonstrates the important role of the duration of contact of bio-NPs with the 30% sucrose solution during UC.

On the surface of photomodified EVs and LPs (Figure 7j–o) isolated on a sucrose cushion we did not find protein corona structures similar to those observed after UC alone (Figure 4). At the same time, an electron-dense material was observed on the surface of chylomicrons, which varied in thickness and was partially fragmented (Figure 7p–t). The chylomicron envelope exhibits a very high electron density (Figure 7r,t), and its appearance clearly differs from that in control samples (Figure 7i). The chylomicron in Figure 7s exhibits another pattern: Its envelope preserved a low electron density, and an electron-dense material is visible on the envelope’s outside. Thus, the isolation of bio-NPs from photomodified FBS and NBS on a sucrose cushion revealed the signs of a protein corona (electron-dense material) on chylomicrons and the absence of such signs on LPs and EVs. The obtained results indicate that incubation in 30% sucrose and washing (90 min with UC at 100,000*g*) deprived photomodified bio-NPs of the ability to preserve the protein corona.

The study presented in this article is a pioneering one. We used the photomodification method that we developed earlier, which allowed us to obtain and visualize a complete protein corona on model NPs with lipid envelope [23]. Using TEM, we tested the applicability of this method for fixing a “natural” corona on serum bio-NPs. FBS and NBS were photomodified, and bio-NPs were isolated by routine methods used for the isolation of bio-NPs. A protein corona was detected in TEM on bio-NPs isolated by UC; its structural variants are presented in the article. Isolation of bio-NPs on a sucrose gradient (12%, 30%, and 50%) did not preserve the protein corona on bio-NPs and resulted in disruption of their ultrastructure. Isolation by a sucrose cushion did not ensure the preservation of the protein corona on EVs and LPs, but it preserved the ultrastructure of the bio-NPs. The protein corona was detected on chylomicrons isolated on the sucrose cushion.

The obtained results demonstrate the possibility to obtain bio-NPs bearing a “natural” protein corona by photomodification of sera and to isolate these particles using a routine UC. The revealed negative effects of prolonged incubation of bio-NPs in 30% sucrose during UC obviously require further studies since this method is widely used in laboratory practice.

## Conclusion

We tested, using TEM, the applicability of the photomodification method for fixing the “natural” protein corona on serum bio-NPs. First, we photomodified FBS and NBS; then, we isolated bio-NPS by routine methods used for the isolation of EVs and LPs. We applied three methods of bio-NP isolation from intact and photomodified FBS and NBS and analyzed the ultrastructure of the resulting particles. The obtained results allowed us to conclude that the “natural” protein corona is present on EVs and LPs; it can be fixed by the photomodifier and visualized by TEM. Isolation on a sucrose gradient (12%, 30% and 50%) did not lead to a positive result; the protein corona on the bio-NPs was lost during the isolation process. Long-term exposure of bio-NPs to 30% sucrose at 100,000*g* led to the disruption of their ultrastructure. Isolation on a sucrose cushion did not preserve the protein corona on EVs and LPs, but it did not damage their ultrastructure. In the samples obtained by this method, chylomicrons bearing a protein corona were found. Thus, for the first time we have obtained images of a “natural” protein corona on natural bio-NPs of blood serum.

## Materials and Methods

For the protein corona studies, we used bio-NPs naturally existing in FBS and NBS, both from Thermo Fisher Scientific (Waltham, MA, USA). Blood serum contains structured components (bio-NPs) and various structureless components, including proteins. Serum samples for TEM examination must be diluted, otherwise an electron-dense “felt” covers the grid, in which nothing can be seen. We used 10% sera because this is the concentration of serum used in cell cultivation, and we plan to study the protein corona on bio-NPs in culture medium in the future. The sera were diluted with 0.01 M phosphate buffer (PBS), pH 7.4, from Sigma-Aldrich (USA).

Prior to the experiments, all serum samples were processed to remove protein aggregates (Figure 2a), as recommended earlier [36]: 10% FBS or NBS was sonicated for 4 min at 30 W, using a Sonorex Digitec DT-31 (Bandelin Electronic, Germany) and then centrifuged at 16000*g* for 4 min (Centrifuge 5415 R, Eppendorf, Germany, rotor F-45-24-11) at room temperature. The supernatant was collected and immediately incubated with PACL (Figure 2b), or used to prepare the corresponding control samples.

To carry out photomodification of the sera, we used a previously developed protocol [23]. In brief, 2 mL of 10% FBS or NBS were mixed with 1.14 μL 0.5 M PACL (the photoactivatable cross-linker 4-azido-*N*-[3-[3-(2,5-dioxopyrrol-1-yl)propanoylamino]propyl]-2-nitrobenzamide, which was synthesized at ICBFM SB RAS, Novosibirsk, Russia) in DMSO. The mixture was incubated at 20 °C under constant stirring on a Thermo-Shaker TS-100C (Biosan, Latvia) at 700 rpm for 2 h. To bind PACL-modified serum proteins to the surface of bio-NPs, the sera were exposed to UV light with a wavelength of 310 nm and 8.2 mW/cm^2^ for 1 min. Sera that were not photomodified served as a control.

Thus, we obtained FBS and NBS samples containing bio-NPs carrying proteins bound by the photomodifier to the particle surface (probable protein corona). Obviously, in addition to bio-NPs, the serum samples contain structureless components, which can significantly complicate the visualization of bio-NPs bearing a protein corona. Our next task was to isolate bio-NPs from these samples and to analyze their surface regarding the presence of a protein corona in TEM.

### Isolation of bio-NPs using ultracentrifugation

UC is still a gold standard for the isolation of EVs from biological fluids [11,25], and we started the isolation of corona-bearing bio-NPs using this method (Figure 2d1). The same procedure was used to isolate bio-NPs from intact sera. 1 mL of 10% photomodified FBS or NBS was mixed with 3 mL of PBS, sonicated for 3 min, and underwent UC for 2 h at 4 °C and 100,000*g* (Ultracentrifuge L8-70M, rotor SW-60, Beckman, USA). The supernatant was removed, the pellet (≈50 μL) was resuspended using a pipette. The obtained samples were applied onto grids to prepare samples for TEM studies. The isolation of bio-NPs was repeated three times. For the second UC (Figure 2d2), the pellet obtained as described above (≈50 μL) was resuspended in 4 mL PBS and underwent UC again under the same conditions. The supernatant was removed, the 50 μL pellet was resuspended and used for TEM studies of bio-NPs. Bio-NPs isolated as described above from intact FBS or NBS were used as controls. The isolation was repeated four times, and all obtained samples were studied in TEM.

### Isolation of bio-NPs using sucrose gradient

Various modifications of the sucrose gradient technique are widely used for the isolation of EVs from biological fluids, and we applied the version of [37]. In 14 mL Ultraclear^TM^ tubes (Beckman Coulter, USA), 3.5 mL portions of 50% (1.46 M), 30% (0.87 M), and 12% (0.35 M) sucrose solution in PBS were successively layered (Figure 2d3). Then, 2 mL of 10% intact FBS or NBS were applied on top of the resulting gradient and underwent UC for 18 h at 4 °C (Ultracentrifuge L8-70M, rotor SW-40, 100,000*g*). The layers were then separated; each was diluted with PBS to 14 mL and underwent UC again for 2 h, to clear from sucrose. Each supernatant was removed, and the pellets were resuspended in 50 µL of remaining supernatant. The grids were prepared from samples of each sucrose fraction and examined in a TEM.

We also ultracentrifuged 12%, 30%, and 50% sucrose solutions without serum and examined the prepared samples in TEM. FBS or NBS were photomodified (see above), and then the sera were purified in the same sucrose gradient. Isolation of bio-NPs from FBS was repeated for three times, and from NBS for four times; all obtained samples were studied in TEM.

### Isolation of bio-NPs using sucrose cushion

The sucrose cushion technique is also widely used in EV studies in different versions [25], and we applied the one described in [35]. In brief, 4 mL of 30% sucrose solution in PBS were placed in 14 mL Ultraclear^TM^ tubes (Beckman Coulter, USA), and 8 mL of 10% intact FBS or NBS were applied on the top (Figure 2d4). The tubes underwent UC for 90 min at 4 °C (Ultracentrifuge L8-70M, rotor SW-40, 100,000*g*). Then, the upper layer was removed, and the bottom layer containing bio-NPs was diluted with PBS to 13.5 mL and thoroughly mixed. The resulting solution underwent UC for 90 min to clear from sucrose, the supernatant was removed, the pellet was resuspended in 50 μL of remaining supernatant, and the samples were prepared for TEM examination. Photomodified FBS or NBS samples containing bio-NPs were purified in the same way.

### Transmission electron microscopy

The size of bio-NPs determines the leading role of TEM in their study since this method is the only method for direct visualization of submicrometer structures. We used negative staining of bio-NP samples, which allows one to see the particles and study their structure, as well as to assess the degree of contamination of the sample with impurities. The technical capabilities of modern TEMs allow for the measurement of object sizes with high accuracy, directly on the computer monitor of a digital camera. Determining the sizes is important for identifying the types of LPs and EVs. TEM was the main method of our study, the aim of which was to visualize the protein corona on bio-NPs. We studied samples of bio-NPs isolated by means of (1) single and (2) double UC, (3) on a sucrose gradient and (4) on a sucrose cushion, more specifically, (1, 2) samples of bio-NPs isolated from photomodified and native FBS and NBS, (3) samples of bio-NPs isolated from photomodified and native FBS and NBS plus samples of sucrose fractions without sera (additional control), and (4) samples of bio-NPs isolated from photomodified and native FBS and NBS.

Negative staining with 0.5% aqueous uranyl acetate solution was performed identically for all samples, using pre-prepared copper grids covered with formvar film. A drop of a sample was adsorbed for 1 min on a grid, excess of the liquid was removed by a pipette; then, the wet grid was placed on a drop of 0.5% aqueous uranyl acetate solution for 10 s, excess liquid was removed with filter paper. Grids were air-dried and examined using a JEM-1400 transmission electron microscope (JEOL, Tokyo, Japan), and digital images were collected using a Veleta digital camera (EM SIS, Münster, Germany). All measurements were made using the iTEM software version 5.2 (EM SIS, Münster, Germany).

## Data Availability

Data generated and analyzed during this study is available from the corresponding author upon reasonable request.

## References

[1] Barz M, Parak W J, Zentel R (2024). Adv Sci.

[2] Nienhaus K, Nienhaus G U (2023). Small.

[3] Zhao T, Ren M, Shi J, Wang H, Bai J, Du W, Xiang B (2024). Biomed Pharmacother.

[4] Blume J E, Manning W C, Troiano G, Hornburg D, Figa M, Hesterberg L, Platt T L, Zhao X, Cuaresma R A, Everley P A (2020). Nat Commun.

[5] Ghebosu R E, Pendiuk Goncalves J, Wolfram J (2024). Nano Lett.

[6] Weber C, Simon J, Mailänder V, Morsbach S, Landfester K (2018). Acta Biomater.

[7] Mohammad-Beigi H, Hayashi Y, Zeuthen C M, Eskandari H, Scavenius C, Juul-Madsen K, Vorup-Jensen T, Enghild J J, Sutherland D S (2020). Nat Commun.

[8] García-Álvarez R, Vallet-Regí M (2021). Nanomaterials.

[9] Kruszewska J, Zajda J, Matczuk M (2021). Talanta.

[10] Önal Acet B, Gül D, Stauber R H, Odabaşı M, Acet Ö (2024). Nanomaterials.

[11] Heidarzadeh M, Zarebkohan A, Rahbarghazi R, Sokullu E (2023). Cell Commun Signaling.

[12] Liam-Or R, Faruqu F N, Walters A, Han S, Xu L, Wang J T-W, Oberlaender J, Sanchez-Fueyo A, Lombardi G, Dazzi F (2024). Nat Nanotechnol.

[13] Dietz L, Oberländer J, Mateos‐Maroto A, Schunke J, Fichter M, Krämer‐Albers E-M, Landfester K, Mailänder V (2023). J Extracell Vesicles.

[14] Tóth E Á, Turiák L, Visnovitz T, Cserép C, Mázló A, Sódar B W, Försönits A I, Petővári G, Sebestyén A, Komlósi Z (2021). J Extracell Vesicles.

[15] Wolf M, Poupardin R W, Ebner‐Peking P, Andrade A C, Blöchl C, Obermayer A, Gomes F G, Vari B, Maeding N, Eminger E (2022). J Extracell Vesicles.

[16] Nguyen P H D, Le A H, Pek J S Q, Pham T T, Jayasinghe M K, Do D V, Phung C D, Le M T N (2022). J Extracell Biol.

[17] Grigor’eva A E, Dyrkheeva N S, Bryzgunova O E, Tamkovich S N, Chelobanov B P, Ryabchikova E I (2017). Biochemistry (Moscow) Suppl Ser B: Biomed Chem.

[18] Bryzgunova O E, Zaripov M M, Skvortsova T E, Lekchnov E A, Grigor’eva A E, Zaporozhchenko I A, Morozkin E S, Ryabchikova E I, Yurchenko Y B, Voitsitskiy V E (2016). PLoS One.

[19] Burkova E E, Grigor’eva A E, Bulgakov D V, Dmitrenok P S, Vlassov V V, Ryabchikova E I, Sedykh S E, Nevinsky G A (2019). Int J Mol Sci.

[20] Sedykh S E, Purvinish L V, Monogarov A S, Burkova E E, Grigor'eva A E, Bulgakov D V, Dmitrenok P S, Vlassov V V, Ryabchikova E I, Nevinsky G A (2017). Biochim Open.

[21] Tupitsyna A V, Grigorieva A E, Soboleva S E, Maltseva N A, Sedykh S E, Poletaeva J, Dmitrenok P S, Ryabchikova E I, Nevinsky G A (2023). Int J Mol Sci.

[22] Simonsen J B, Münter R (2020). Angew Chem, Int Ed.

[23] Epanchintseva A V, Poletaeva J E, Bakhno I A, Belov V V, Grigor’eva A E, Baranova S V, Ryabchikova E I, Dovydenko I S (2023). Nanomaterials.

[24] Vance D E, Vance J E (2008). Biochemistry of Lipids, Lipoproteins and Membranes.

[25] Langevin S M, Kuhnell D, Orr-Asman M A, Biesiada J, Zhang X, Medvedovic M, Thomas H E (2019). RNA Biol.

[26] Gao Y, Huang Y, Ren C, Chou P, Wu C, Pan X, Quan G, Huang Z (2024). J Mater Chem B.

[27] Ranjan P, Verma S K, Di Nardo P, Dhingra S, Desiderio V (2024). Exosomes Isolation, Purification, and Characterization. Adult Stem Cells.

[28] Gu M, Chen P, Zeng D, Jiang X, Lv Q, Li Y, Zhang F, Wan S, Zhou Q, Lu Y (2023). Cell Commun Signaling.

[29] van de Vlekkert D, Qiu X, Annunziata I, d'Azzo A (2020). Bio-Protoc.

[30] Kim N-H, Kim J, Lee J-Y, Bae H-A, Kim C Y (2023). Nutrients.

[31] Yao M, Chen J, Zheng J, Song M, McClements D J, Xiao H (2013). Food Funct.

[32] Nauli A M, Sun Y, Whittimore J D, Atyia S, Krishnaswamy G, Nauli S M (2014). Physiol Rep.

[33] Anderson L J, Boyles J K, Hussain M M (1989). J Lipid Res.

[34] Ly H L, Mortimer B C, Baker E, Redgrave T G (1992). Biochem J.

[35] Gupta S, Rawat S, Arora V, Kottarath S K, Dinda A K, Vaishnav P K, Nayak B, Mohanty S (2018). Stem Cell Res Ther.

[36] Santelices J, Ou M, Hui W W, Maegawa G H B, Edelmann M J (2022). Bio-Protoc.

[37] Smyth T, Kullberg M, Malik N, Smith-Jones P, Graner M W, Anchordoquy T J (2015). J Controlled Release.

